# Multiple primary and familial melanoma as distinct high-risk phenotypes: a retrospective cohort study

**DOI:** 10.3389/fgene.2026.1846948

**Published:** 2026-07-31

**Authors:** Andrea Carugno, Giovanni Paolino, Mario Valenti, Stefano Bighetti, Vincenzo Maione, Noemi Brigenti, Lorenza Bertù, Nicola Zerbinati

**Affiliations:** 1 Department of Medicine and Surgery, University of Insubria, Varese, Italy; 2 Dermatology Unit, Ospedale di Circolo Fondazione Macchi, Varese, Italy; 3 Unit of Dermatology, IRCCS Ospedale San Raffaele, Milan, Italy; 4 Faculty of Medicine and Surgery, Università Vita-Salute San Raffaele, Milan, Italy; 5 Dermatology Unit, IRCCS Humanitas Research Hospital, Rozzano, Italy; 6 Department of Biomedical Sciences, Humanitas University, Milan, Italy; 7 Dermatology Department, University of Brescia, ASST Spedali Civili di Brescia, Brescia, Italy; 8 Section of Dermatology and Venereology, Department of Medicine, University of Verona, Verona, Italy; 9 Department of Medicine and Innovation Technology (DiMIT), University of Insubria, Varese, Italy

**Keywords:** cutaneous melanoma, environmental exposure, familial melanoma, genetic susceptibility, high-risk phenotype, multiple primary melanoma

## Abstract

**Introduction:**

Patients with multiple primary melanoma (MPM) and familial melanoma (FM) are commonly grouped as a single high-risk category, although they may represent biologically distinct entities reflecting different contributions of genetic susceptibility and environmental exposure. The aim of this study was to compare clinical, phenotypic, environmental, histopathological, and genetic features among patients with MPM only, FM only, and combined MPM+FM, as well as to identify independent predictors of MPM phenotype.

**Methods:**

In this retrospective observational study, 333 high-risk melanoma patients were stratified into MPM only, FM only, and MPM+FM. Clinical and histopathological variables were analyzed using appropriate statistical tests. Univariate and multivariate logistic regression models were used to identify independent predictors of MPM, comparing patients with MPM only versus those with FM only.

**Results:**

Age at first melanoma diagnosis differed significantly across groups (p < 0.001), with MPM patients presenting older age compared with FM and MPM+FM. Early-onset melanoma (<40 years) was more frequent in FM (40.7%) and MPM+FM (43.1%) than in MPM (21.0%; p = 0.001). A family history of melanoma-associated cancers was observed exclusively in FM and MPM+FM (p < 0.001). Tanning bed use was more frequent in patients with a familial component (p = 0.02). Histopathological features and nevus burden were comparable across groups. In both univariate and multivariate logistic regression analyses, increasing age (OR 1.04, 95% CI 1.02–1.07; p = 0.003) and male sex (OR 2.90, 95% CI 1.41–5.99; p = 0.004) were independently associated with MPM.

**Discussion:**

MPM and FM represent distinct high-risk melanoma phenotypes. MPM is associated with older age and male sex, suggesting a predominant role of cumulative environmental exposure, whereas FM is characterized by earlier onset and broader cancer susceptibility. These findings support phenotype-driven risk stratification and tailored surveillance strategies.

## Introduction

Cutaneous melanoma represents a major and growing public health concern, with incidence rates increasing more rapidly than those of most other solid tumors worldwide (Gandini et al., 2005; Moore et al., 2015; Wang et al., 2025). While most melanoma cases arise sporadically and are largely driven by environmental factors (such as ultraviolet [UV] radiation exposure) along with host-related characteristics such as fair skin phenotype and high nevus burden, a clinically relevant subset of patients exhibits features suggestive of inherited susceptibility (Soura et al., 2016; Rossi et al., 2019; Zocchi et al., 2021). Melanoma development is increasingly understood as the result of a complex interplay between genetic predisposition and environmental exposure, a concept formalized in the divergent pathway model, which proposes distinct biological routes leading to melanoma, including a nevus-driven pathway associated with genetic susceptibility and an alternative pathway driven by cumulative UV-induced damage (Whiteman et al., 2003). Familial melanoma (FM) is generally defined by the occurrence of melanoma in multiple family members, including at least one first- or second-degree relative, reflecting a pattern suggestive of inherited susceptibility rather than purely sporadic disease, and it accounts for approximately 5%–15% of all melanoma cases (Soura et al., 2016; Bishop et al., 2002). Despite this relatively small proportion, FM represents a critical model for understanding the interplay between genetic predisposition and environmental triggers in melanoma pathogenesis.

The genetic landscape of hereditary melanoma is complex and only partially elucidated. Germline pathogenic variants in high-penetrance genes, particularly *CDKN2A* and, less frequently, *CDK4*, are identified in approximately 20%–40% of melanoma-prone families (Bishop et al., 2002; Soura et al., 2016; Bruno et al., 2022; Primiero et al., 2024). In recent years, advances in next-generation sequencing technologies have broadened the spectrum of candidate susceptibility genes, including *BAP1*, *POT1*, *TERT*, *ACD*, *TERF2IP*, and *MITF,* as well as additional intermediate- and low-penetrance genes such as *MC1R*, *ATM*, and *CHEK2* (Casula et al., 2019; Ribeiro Moura Brasil Arnaut et al., 2021; Goldstein et al., 2023; Hayward et al., 2017). However, a substantial proportion of familial melanoma cases remains genetically unexplained, suggesting the involvement of polygenic inheritance models, gene–environment interactions, or yet unidentified genetic determinants (Casula et al., 2019; Ribeiro Moura Brasil Arnaut et al., 2021). This incomplete genetic characterization underscores the continued importance of clinical phenotyping in the identification and management of high-risk individuals.

A key clinical manifestation associated with increased melanoma susceptibility is the development of multiple primary melanomas (MPM). The reported incidence of MPM varies across populations, ranging from approximately 1% to over 8%, depending on geographic, environmental, and surveillance-related factors (Moore et al., 2015; De Summa et al., 2020). Patients with MPM are known to have a higher likelihood of harboring germline pathogenic or likely pathogenic variants compared with those with a single primary melanoma, particularly in the presence of a positive family history (Soura et al., 2016; Zocchi et al., 2021). However, MPM is not exclusively linked to hereditary predisposition. In many cases, it may arise in the absence of identifiable genetic alterations, likely reflecting the cumulative effect of environmental exposures, especially chronic UV radiation, together with individual susceptibility factors (Zocchi et al., 2021; Ferrone et al., 2005). Consequently, MPM represents a clinically heterogeneous phenotype that may encompass both genetically driven and environmentally driven pathways.

Despite these differences, patients with MPM and those with FM are frequently grouped together as a single “high-risk melanoma” category in both clinical practice and research settings. While this classification is useful for identifying individuals requiring intensified surveillance, it may obscure important biological and clinically relevant distinctions between these subgroups. In particular, the relative contribution of inherited susceptibility versus cumulative environmental damage may differ substantially between patients who develop multiple melanomas without a family history and those with familial aggregation who may present with a single primary tumor. Disentangling these components is essential for improving risk stratification, optimizing follow-up strategies, and refining indications for genetic counseling and testing.

Emerging evidence suggests that age at onset, sex distribution, nevus burden, and patterns of UV exposure may differ between patients with MPM and those with FM, supporting the hypothesis of partially divergent pathogenic pathways. In a previous analysis of our cohort, we observed significant age-related differences in clinical presentation, with older patients more frequently developing multiple primary melanomas and younger individuals more commonly exhibiting a familial phenotype and a higher nevus burden (Carugno et al., 2025a). These findings highlight the need for a more granular characterization of high-risk melanoma populations beyond traditional binary classifications.

To date, few studies have directly compared patients with multiple primary melanoma and those with familial melanoma within the same cohort using a comprehensive phenotypic and clinical approach. As a result, the extent to which these entities represent distinct biological and clinical phenotypes remains insufficiently characterized.

Building upon this background, the present study aims to systematically investigate the heterogeneity of high-risk melanoma by stratifying patients into three distinct groups: those with multiple primary melanomas without familial history (MPM only), those with familial melanoma with a single primary tumor (FM only), and those presenting both features (MPM+FM). By comparing demographic, phenotypic, environmental, histopathological, and genetic characteristics across these subgroups, we seek to delineate their clinical profiles and identify potential distinguishing features.

Furthermore, to better understand the determinants of melanoma multiplicity independent of familial aggregation, we performed univariate and multivariate logistic regression analyses comparing patients with MPM only to those with FM only. This approach allows for the identification of independent factors associated with the development of multiple primary melanomas, providing insight into the relative contribution of intrinsic versus extrinsic risk factors. Ultimately, a more refined understanding of these phenotypes may support the development of personalized surveillance strategies and more targeted approaches to risk assessment in patients with high-risk melanoma.

## Materials and methods

This observational, retrospective, cross-sectional study was conducted at the Melanoma Clinic of Bergamo Hospital, Italy. The study population included 333 consecutive patients evaluated at our center between 2021 and 2024, selected from a larger cohort of melanoma patients under follow-up. Inclusion criteria were defined as the presence of MPM, characterized by two or more histologically confirmed primary melanomas (synchronous or metachronous), and/or FM, defined as a diagnosis of cutaneous melanoma in the proband and at least one first- or second-degree relative. Patients were stratified into three mutually exclusive groups according to their clinical presentation, including individuals with multiple primary melanomas without a family history (MPM only), patients with a single primary melanoma and a positive family history (FM only), and those presenting both multiple primary melanomas and familial aggregation (MPM+FM). All patients were consecutively included to minimize selection bias.

A comprehensive clinical history was collected for each patient. Demographic variables included age at diagnosis, sex, place of birth and residence, body mass index, education level, marital status, and occupation. Phenotypic characteristics included Fitzpatrick skin phototype, eye color, and hair color at the age of 20 years. Sun exposure patterns were assessed based on the history of blistering childhood sunburns, occupational ultraviolet exposure, use of artificial tanning devices (defined as current or past use for at least 6 months), and sunscreen use. A quantitative assessment of melanocytic nevi was performed by estimating the total number of nevi and arbitrarily calculating nevus density as the number of nevi per square meter. Body surface area was determined using the Mosteller formula to normalize nevus counts. Family history of melanoma was recorded in detail, including the number of affected relatives, degree of relationship, age at diagnosis, and the presence of melanoma-associated malignancies, such as pancreatic, renal, or pleural and peritoneal mesotheliomas. The selection of these specific associated tumors was strictly based on the formal inclusion criteria for genetic counseling and testing established by the Italian Melanoma Intergroup (IMI) (Instructions for Genetic Teleconsultation, 2026). Importantly, these associated malignancies were recorded exclusively if they segregated within the same branch of the family affected by melanoma. This oncological family history was self-reported by the patients during the clinical interview. Information on known germline variants predisposing to melanoma was recorded when available for patients who had already undergone genetic testing prior to our evaluation.

Histopathological data were retrieved from pathology reports for all primary melanomas and included histological subtype, Breslow thickness, ulceration status, mitotic rate, and the presence of histological regression. Tumor staging was defined according to the 8th edition of the American Joint Committee on Cancer (AJCC) staging system. The severity of the first melanoma was categorized based on the TNM classification at diagnosis as follows: ‘Low’ severity included *in situ* melanomas (pTis); ‘Intermediate’ severity included pT1a tumors; and ‘High’ severity encompassed pT1b and all higher T categories (pT2a–pT4b), as well as any case presenting with regional lymph node involvement (N+) or distant metastasis (M+) at the time of diagnosis.

Genetic tele-counseling and germline testing were offered to eligible patients in collaboration with the Unit of Genetics of Rare Cancers at IRCCS Ospedale Policlinico San Martino (Genoa, Italy), following the criteria established by the Italian Melanoma Intergroup (Carugno et al., 2025b; Instructions for Genetic Teleconsultation, 2026). Germline DNA was extracted from peripheral blood leukocytes, and mutational analysis was performed using a next-generation sequencing multigene panel targeting high- and intermediate-penetrance melanoma susceptibility genes, including *CDKN2A*, *CDK4*, *BAP1*, *POT1*, *ACD*, *TERF2IP*, and *MITF*. Variant classification was performed in accordance with the most recent international guidelines (Richards et al., 2015; Durkie et al., 2024), and only pathogenic or likely pathogenic variants were considered for genotype–phenotype correlation analyses, while variants of uncertain significance were excluded.

Descriptive statistics were used to summarize baseline characteristics across the study groups. Continuous variables were expressed as mean ± standard deviation or median and interquartile range, depending on data distribution, while categorical variables were presented as frequencies and percentages. Comparisons between groups were performed using the chi-square test or Fisher’s exact test for categorical variables and one-way ANOVA or Kruskal–Wallis tests for continuous variables, as appropriate. To identify independent predictors of melanoma multiplicity, logistic regression analyses were performed comparing patients with MPM only and those with FM only, in order to isolate factors associated with the development of multiple primary melanomas independently of familial aggregation. Variables with a p-value ≤ 0.05 at univariate analysis were included in a multivariate logistic regression model, and results were reported as odds ratios with 95% confidence intervals. For categorical variables with more than two levels, if at least one dummy category reached the significance threshold, the entire variable was retained in the model. Patients with missing data for variables included in the multivariate model were handled using complete-case analysis. A two-sided p-value < 0.05 was considered statistically significant. All statistical analyses were performed using SAS ODA (version 9.4, SAS Institute Inc., Cary, NC, USA).

The study was conducted in accordance with the principles of the Declaration of Helsinki and was approved by the Ethics Committee of Bergamo (Resolution No. 834, May 24, 2022). Written informed consent was obtained from all participants for the use of anonymized clinical and genetic data.

## Results

The study cohort included 333 patients selected for having multiple primary melanomas and/or familial melanoma from a larger population of 1,305 melanoma patients followed at the Melanoma Clinic. Among them, 164 patients (49.2%) were classified as having FM, 106 (31.8%) as MPM, and 63 (18.9%) as MPM + FM. The mean age at first melanoma diagnosis in the overall cohort was 48.5 years. To allow a more reliable comparison between phenotypic subgroups, comparative analyses were restricted to patients with adequate follow-up, defined as a first melanoma diagnosis on or before June 30, 2019, to ensure a minimum of 5 years of follow-up at the time of patient recruitment. This subgroup included 233 patients, of whom 88 had MPM, 91 had FM, and 54 had MPM+FM.

Significant differences emerged across the three groups (Table 1). Age at first melanoma diagnosis differed significantly according to melanoma type (p < 0.001), with patients in the MPM group being older at diagnosis than those in the FM and MPM+FM groups. Median age at first diagnosis was 53 years in MPM, compared with 43 years in FM and 40 years in MPM+FM. Consistently, early-onset melanoma, defined as diagnosis before 40 years of age, was more frequent in FM (40.7%) and MPM+FM (43.1%) than in MPM (21.0%; p = 0.001).

**TABLE 1:** Demographic, phenotypic, and environmental characteristics of patients with multiple primary melanoma (MPM), familial melanoma (FM), and combined MPM+FM.

Characteristic	MPM (n = 88)	FM (n = 91)	MPM+FM (n = 54)	p-value
Age at first melanoma diagnosis (years)				
Mean ± SD	51.3 ± 13.7	43.1 ± 14.1	42.6 ± 13.3	<0.001*^a^
Median (IQR)	53 (42–62)	43 (31–53)	40 (36–50)	​
Age classes, n (%)				
<40 years	17 (21.0)	37 (40.7)	22 (43.1)	0.001*^b^
40–60 years	39 (48.1)	44 (48.3)	24 (47.1)	​
>60 years	25 (30.9)	10 (11.0)	5 (9.8)	​
Severity of first melanoma, n (%)				
Low	10 (12.5)	18 (20.0)	12 (24.5)	0.18^b^
Intermediate	41 (51.2)	52 (57.8)	24 (49.0)	​
High	29 (36.2)	20 (22.2)	13 (26.5)	​
Family history of related cancers, n (%)				
Yes	0 (0.0)	18 (21.2)	15 (28.3)	<0.001*^c^
No	88 (100.0)	73 (78.8)	39 (71.7)	​
Fitzpatrick skin type, n (%)				
I	8 (10.3)	3 (3.7)	5 (10.2)	0.34^c^
II	44 (56.4)	43 (52.4)	28 (57.1)	​
III	26 (33.3)	36 (43.9)	16 (32.7)	​
Skin color, n (%)				
Fair	52 (66.7)	47 (57.3)	36 (73.5)	0.31^c^
Intermediate	24 (30.8)	31 (37.8)	13 (26.5)	​
Olive	2 (2.6)	4 (4.9)	0 (0.0)	​
Occupational UV exposure, n (%)				
Yes	7 (8.4)	7 (8.4)	8 (15.4)	0.35^b^
No	76 (91.6)	76 (91.6)	44 (84.6)	​
History of sunburns, n (%)				
Adulthood only	7 (9.0)	8 (9.8)	7 (14.6)	0.34^c^
Childhood only	11 (14.1)	15 (18.3)	12 (25.0)	​
Both childhood and adulthood	50 (64.1)	54 (65.9)	27 (56.2)	​
No	10 (12.8)	5 (6.1)	2 (4.2)	​
Use of sunscreen, n (%)				
SPF 30–50+	63 (80.8)	69 (84.1)	40 (85.1)	0.54^c^
SPF <30	0 (0.0)	2 (2.4)	1 (2.1)	​
No	15 (19.2)	11 (13.4)	6 (12.8)	​
Use of tanning beds, n (%)				
Yes	23 (29.5)	40 (48.8)	24 (50.0)	0.02*^b^
No	55 (70.5)	42 (51.2)	24 (50.0)	​
Who noticed the lesion first, n (%)				
Patient themselves	28 (36.8)	34 (42.5)	21 (46.7)	0.44^c^
Dermatologist	29 (38.2)	26 (32.5)	14 (31.1)	​
Spouse	7 (9.2)	5 (6.2)	4 (8.9)	​
Friend/Family member	6 (7.9)	13 (16.2)	3 (6.7)	​
Other specialist/GP	6 (7.9)	2 (2.5)	3 (6.7)	​

Data represent patients diagnosed with their first melanoma by June 30, 2019 (a cut-off date chosen to ensure a minimum follow-up period of 5 years). Categorical variables are reported as frequencies (percentages); continuous variables as means ± standard deviations (SD) and medians (interquartile ranges, IQR). Statistically significant p-values (<0.05) are marked with an asterisk (*). p-value calculated using.

^a^ Kruskal-Wallis test.

^b^ Pearson’s Chi-square test.

^c^ Fisher’s exact test.

Abbreviations: MPM: multiple primary melanoma; FM: familial melanoma; UV: ultraviolet; SPF: sun protection factor; GP: general practitioner.

A family history of melanoma-associated malignancies, including pancreatic, renal, or pleural and peritoneal mesotheliomas, was reported in 21.2% of FM patients and 28.3% of MPM+FM patients, whereas no such history was observed in the MPM group (p < 0.001). Among environmental and behavioral variables, tanning bed use also differed significantly across groups (p = 0.02), being more frequently reported in FM (48.8%) and MPM+FM (50.0%) than in MPM (29.5%). Regarding other demographic, phenotypic, and environmental characteristics, including Fitzpatrick skin type, skin color, occupational UV exposure, history of sunburns, sunscreen use, and the primary detector of the lesion, no statistically significant differences were observed among the three groups (Table 1).

Histopathological, genetic, and nevus-related characteristics were largely comparable across melanoma phenotypes (Table 2). No statistically significant differences were observed in severity of the first melanoma, anatomical site, histological subtype, growth pattern, regression, nevus remnants, mitotic count, total nevus count, atypical nevus count, or nevus density measures. Ulceration tended to be more frequent in MPM patients (17.1%) than in FM (5.7%) and MPM+FM patients (12.8%), although this difference did not reach statistical significance (p = 0.07). Detailed histopathological and nevus-related data are reported in Table 2.

**TABLE 2:** Histopathological and nevus burden characteristics stratified by phenotype.

Characteristic	MPM (n = 88)	FM (n = 91)	MPM+FM (n = 54)	p-value
Primary tumor characteristics				
Anatomical location, n (%)				
Head-neck	3 (3.7)	8 (8.8)	2 (4.1)	0.92^c^
Trunk (Chest, Abdomen, Back)	43 (52.4)	44 (48.4)	21 (42.9)	​
Upper limb	12 (14.6)	11 (12.1)	7 (14.3)	​
Lower limb	22 (26.8)	23 (25.3)	16 (32.7)	​
Other (Foot, Hand, Buttocks)	2 (2.4)	5 (5.5)	3 (6.1)	​
Histological subtype, n (%)				
Superficial spreading	72 (90.0)	82 (91.1)	45 (91.8)	0.52^c^
Nodular/Amelanotic nodular	6 (7.4)	2 (2.2)	1 (2.0)	​
Lentigo maligna	1 (1.2)	3 (3.3)	2 (4.1)	​
Other rare subtypes†	1 (1.2)	3 (3.3)	1 (2.0)	​
Growth phase, n (%)				
Radial	35 (56.4)	57 (71.2)	29 (64.4)	0.19^b^
Vertical	27 (43.5)	23 (28.7)	16 (35.6)	​
Regression, n (%)				
Not identified	45 (69.2)	65 (78.3)	32 (72.7)	0.55^c^
<75% regression	18 (27.7)	14 (16.9)	11 (25.0)	​
>75% regression	2 (3.1)	4 (4.8)	1 (2.3)	​
Ulceration present, n (%)	12 (17.1)	5 (5.7)	6 (12.8)	0.07^b^
Nevus remnants present, n (%)	19 (31.1)	25 (33.3)	19 (45.2)	0.30^b^
Mitotic count, Median (IQR)	0 (0–2)	0 (0–0.9)	0 (0–0.9)	0.10^a^
NEVUS BURDEN and DENSITY‡				
Total nevi, Median (IQR)	87 (41–173)	91 (48–157)	121 (74–172)	0.63^a^
Atypical nevi, Median (IQR)	0 (0–3)	0 (0–3)	1 (0–4)	0.56^a^
% Of atypical nevi, Median (IQR)	0 (0–2.3)	0 (0–2.2)	1.2 (0–3.4)	0.32^a^
Nevus density (nevi/m^2^), Median (IQR)	47.6 (23.3–92.2)	51.4 (27.8–92.2)	70.1 (37.2–96.3)	0.43^a^
Atypical nevus density, Median (IQR)	0 (0–1.5)	0 (0–1.6)	0.6 (0–2.2)	0.50^a^
Nevus density at primary site, Median (IQR)	50.2 (24.4–103.9)	59.9 (34.2–91.8)	70.9 (37.6–109.2)	0.66^a^
Nevus density at secondary site, Median (IQR)§	51.2 (19.6–128.6)	-	51.1 (35.6–115.3)	0.75^a^
Atypical density at primary site, Median (IQR)	0 (0–0)	0 (0–0)	0 (0–3.4)	0.41^a^
Atypical density at secondary site, Median (IQR)§	0 (0–0)	-	0 (0–0)	0.67^a^

Data represent patients diagnosed with their first melanoma by June 30, 2019 (a cut-off date chosen to ensure a minimum follow-up period of 5 years). Categorical variables are frequencies (percentages). Continuous variables for nevus burden are reported as Medians (Interquartile Ranges, IQR) due to their typically non-normal distribution. p-value calculated using.

^a^ Kruskal-Wallis test.

^b^ Pearson’s Chi-square test.

^c^ Fisher’s exact test.

Abbreviations: MPM: multiple primary melanoma; FM: familial melanoma.

† Includes Acral lentiginous, Spitzoid, and Nevoid melanoma subtypes.

‡ Slight variations in denominators for nevus variables are due to missing data for specific counts.

§ Assessed only in patients who developed a second primary melanoma (applicable to MPM, and MPM+FM, groups).

Within the overall study cohort, 192 patients fulfilled the Italian Melanoma Intergroup (IMI) eligibility criteria. Among them, 3 patients had already undergone genetic testing previously, and their genetic status was recorded. Genetic counseling and testing were proposed to the remaining 189 eligible patients; however, 6 of them declined the evaluation. Consequently, 183 patients underwent germline genetic testing during the study period. Overall, genetic data were available for 186 patients (55.9% of the entire cohort). Only Class 4 (likely pathogenic) and Class 5 (pathogenic) variants were considered. The overall diagnostic yield in this tested subgroup was 4.3% (8/186 patients). When stratifying these positive findings by clinical phenotype, the combined MPM+FM group exhibited the highest number of pathogenic variants, with 4 patients carrying *CDKN2A* variants (three Class 5 variants: c.251 A>T (p.Asp84Val) and c.71G>C (p.Arg24Pro); and one Class 4 variant: c.458-1G>C). In the FM-only group, 3 patients tested positive: two carried *CDKN2A* Class 5 variants (c.71G>C (p.Arg24Pro) and c.251 A>T (p.Asp84Val)), and one harbored a *POT1* Class 5 variant (c.258dupT (p.Glu87fs)). Strikingly, only 1 patient in the purely sporadic MPM group was found to carry a pathogenic variant (*CDKN2A* c.301G>T (p.Gly101Trp)).

From a clinical phenotypic perspective, this single mutated patient in the purely sporadic MPM group presented with 3 primary melanomas, with the first diagnosis occurring at 49 years of age. Conversely, patients harboring pathogenic variants in the familial groups (FM and MPM+FM) exhibited a notably earlier onset of disease (median age at first melanoma diagnosis: 32 years, range 22-52 years). Within these familial subgroups, mutated patients presented up to 2 primary melanomas each, and their pedigrees were typically characterized by 1–2 first- or second-degree relatives affected by melanoma or associated malignancies, such as pancreatic cancer. Importantly, in line with classical hereditary syndromes, the vast majority of melanoma diagnoses among these affected relatives (71%) consistently occurred at an early age (≤ 60 years), and in two of these families, the affected relatives themselves exhibited a multiple primary melanoma phenotype.

To identify factors independently associated with melanoma multiplicity, univariate and multivariate logistic regression analysis were performed comparing MPM-only patients with FM-only patients, excluding the MPM+FM group (Table 3). In univariate logistic regression analysis, older age at first melanoma diagnosis was associated with increased odds of MPM (OR 1.04 per year, 95% CI 1.02–1.07; p < 0.001), and male sex was strongly associated with the MPM phenotype (OR 3.23, 95% CI 1.75–5.96; p < 0.001). The use of artificial tanning devices (current or past use for at least 6 months) was inversely associated with MPM in the univariate model (OR 0.44, 95% CI 0.23–0.84; p = 0.01), whereas high severity of the first melanoma showed a borderline association (OR 2.61, 95% CI 1.00–6.82; p = 0.05).

**TABLE 3:** Univariate and multivariate logistic regression models identifying factors associated with the Multiple Primary Melanoma (MPM) phenotype compared to Familial Melanoma (FM).

Variable	Univariate analysis	​	Multivariate analysis	​
	OR (95% CI)	*p*-value	OR (95% CI)	*p*-value
Age at first melanoma diagnosis	1.04 (1.02–1.07)	<0.001*	1.04 (1.02–1.07)	0.003*
Gender				
Female	1.00 (Ref)	​	1.00 (Ref)	​
Male	3.23 (1.75–5.96)	<0.001*	2.90 (1.41–5.99)	0.004*
Fitzpatrick skin type				
Type I	1.00 (Ref)	​	-	-
Type II	0.38 (0.10–1.54)	0.18	-	-
Type III	0.27 (0.06–1.12)	0.07	-	-
Occupational UV exposure (Yes vs no)	1.00 (0.33–2.99)	0.99	-	-
Use of tanning beds (Yes vs no)	0.44 (0.23–0.84)	0.01*	0.64 (0.30–1.35)	0.24
Childhood sunburns (Yes vs no)	0.72 (0.37–1.39)	0.33	-	-
Location of first melanoma				
Head/Neck	1.00 (Ref)	​	-	-
Extremities	2.59 (0.63–10.58)	0.18	-	-
Trunk	2.50 (0.62–10.01)	0.20	-	-
Severity of first melanoma				
Low	1.00 (Ref)	​	1.00 (Ref)	​
Intermediate	1.42 (0.59–3.40)	0.43	1.43 (0.52–3.94)	0.49
High	2.61 (1.00–6.82)	0.05*	1.54 (0.50–4.71)	0.45

The combined phenotype group (Multiple + Familial Melanoma) was excluded from this specific analysis to directly compare pure MPM, and FM., Variables with a p-value ≤0.05 in the univariate analysis were included in the multivariate model. An Odds Ratio (OR) > 1 indicates a higher likelihood of being in the Multiple Primary Melanoma (MPM) group compared to the Familial Melanoma (FM) group. Statistically significant p-values (≤0.05) are marked with an asterisk (*). p-value calculated using the Wald Chi-square test for logistic regression.

Abbreviations: OR: odds ratio; CI: confidence interval; Ref: Reference category; UV: ultraviolet.

**FIGURE 1:**
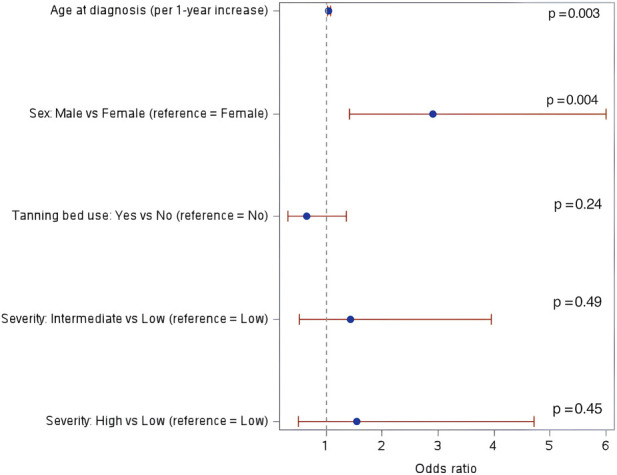
Forest plot of multivariate logistic regression analysis showing independent predictors of multiple primary melanoma (MPM) compared with familial melanoma (FM), with odds ratios (ORs) and 95% confidence intervals (CIs).

In the multivariate logistic regression model, only age at first melanoma diagnosis and male sex remained independently associated with MPM. Age retained a significant effect (adjusted OR 1.04, 95% CI 1.02–1.07; p = 0.003), and male sex remained a strong independent predictor (adjusted OR 2.90, 95% CI 1.41–5.99; p = 0.004). The inverse association observed for tanning bed use in univariate analysis was attenuated and no longer significant after adjustment (adjusted OR 0.64, 95% CI 0.30–1.35; p = 0.24), and the association between high first-melanoma severity and MPM also lost significance after adjustment (adjusted OR 1.54, 95% CI 0.50–4.71; p = 0.45). A graphical representation of the multivariate model is shown in Figure 1.

## Discussion

This study demonstrates that high-risk melanoma comprises clinically and biologically distinct phenotypes, rather than a single homogeneous entity, by differentiating between MPM, FM, and the combined MPM+FM phenotype. Our findings support the concept that these subgroups might represent partially distinct entities, driven by different contributions of genetic susceptibility and environmental exposure, in line with the divergent pathway model of melanoma development (Whiteman et al., 2003).

A key observation of this study is the significant difference in age at first melanoma diagnosis across the analyzed samples. Patients with FM and MPM+FM presented at a younger age compared with those with MPM only, reinforcing the role of inherited susceptibility in accelerating disease onset. In contrast, patients with sporadic MPM were older at diagnosis, supporting the hypothesis that cumulative environmental exposure, particularly ultraviolet radiation, plays a dominant role in this subgroup. These findings are consistent with previous evidence suggesting that melanoma development may follow distinct biological pathways depending on the interplay between genetic background and environmental factors (Whiteman et al., 2003; Armstrong and Kricker, 2001).

The distribution of melanoma-associated malignancies further supports this distinction. A positive family history of cancers such as pancreatic or renal tumors was observed exclusively in FM and MPM+FM patients, suggesting a broader cancer susceptibility phenotype in these groups. This has relevant clinical implications, as it highlights the need for a more comprehensive risk assessment that extends beyond melanoma in patients with familial aggregation, as already emphasized in studies on hereditary melanoma syndromes (Soura et al., 2016).

Behavioral factors also differed across subgroups. The higher prevalence of tanning bed use (current or past use for at least 6 months) in FM and MPM+FM patients may reflect generational or behavioral patterns, but it also raises the possibility that artificial ultraviolet exposure may act as a disease accelerator in genetically predisposed individuals. However, this association should be interpreted with caution, as it did not retain significance in multivariate analysis, and may reflect confounding effects related to age and exposure patterns rather than a direct causal relationship (Armstrong and Kricker, 2001).

The logistic regression analysis provides further insight into the determinants of melanoma multiplicity. Age at diagnosis and male sex emerged as the only independent predictors of multiple primary melanomas when compared with familial melanoma. The strong association with male sex is consistent with previous studies showing sex-related differences in melanoma incidence and outcomes (Joosse et al., 2012). This finding may reflect differences in cumulative sun exposure, behavioral factors, or underlying biological susceptibility. Importantly, the persistence of age as an independent factor supports the concept that melanoma multiplicity, in the absence of familial aggregation, is largely driven by time-dependent exposure rather than inherited risk.

In contrast, variables such as tanning bed use and severity of the first melanoma, although significant or borderline in univariate analysis, lost significance after adjustment, suggesting that these factors are likely confounded by demographic variables rather than acting as independent drivers of multiple melanoma development. Similarly, no significant associations were observed for phenotypic characteristics, ultraviolet exposure variables, or genetic test results.

Interestingly, the diagnostic yield of germline testing for classical high-penetrance melanoma susceptibility genes was relatively low across the overall cohort, with pathogenic variants primarily concentrated in patients with a familial component. However, this finding is in line with recent Italian multicenter and single-center studies, which have demonstrated a significantly lower prevalence of pathogenic variants in high-risk patients (ranging from approximately 9%–11% using multigene panels) compared to less recent literature (Bruno et al., 2022; Ferrara et al., 2024). The observation that only one patient in the purely sporadic MPM group harbored a pathogenic variant (*CDKN2A*) suggests that the MPM-only phenotype might be less frequently driven by classic high-penetrance Mendelian inheritance compared to familial cases (Hayward et al., 2017). This limited mutational burden in sporadic MPM brings attention to the concept of “missing heritability” often discussed in high-risk melanoma (Dalmasso and Ghiorzo, 2020). Rather than being triggered exclusively by single high-penetrance variants, the genetic susceptibility in pure MPM patients could be polygenic. It may result from the additive effect of multiple low-to-intermediate penetrance variants interacting with cumulative environmental damage. Our multivariate model, which identified older age and male sex as the only independent predictors of the MPM phenotype, seems to support this hypothesis. These demographic variables are well-known proxies for prolonged cumulative ultraviolet (UV) exposure (Armstrong and Kricker, 2001). Thus, sporadic MPM might represent a clinical manifestation of a gene-environment interaction, where a moderate polygenic background requires prolonged environmental exposure to manifest as multiple tumors (Whiteman et al., 2003). In fact, recent studies have demonstrated that Polygenic Risk Scores (PRS) are significantly higher in patients with multiple primary melanomas compared to those with single melanomas, confirming a strong polygenic basis for the MPM phenotype (Potjer et al., 2021; Pellegrini et al., 2024). Conversely, the earlier onset of disease, broader cancer susceptibility, and slightly higher mutational yield observed in the FM and MPM+FM groups appear more closely aligned with classical hereditary tumor syndromes. (Soura et al., 2016; Rossi et al., 2019). However, it must be acknowledged that the significantly younger age at melanoma diagnosis in these familial could be influenced by a surveillance bias, as close relatives of affected patients routinely undergo earlier and more frequent dermatological screenings compared to purely sporadic cases. Altogether, these results indicate that current multigene panels, which largely focus on high-penetrance genes, may have limited explanatory power for the purely sporadic MPM phenotype (Ribeiro Moura Brasil Arnaut et al., 2021; Dalmasso and Ghiorzo, 2020). In the future, diagnostic and risk-stratification strategies for these specific patients might benefit from the implementation of PRS, which are increasingly proving useful in improving melanoma risk prediction and capturing the complex genetic architecture underlying environmentally driven multiple tumors (Wong et al., 2023; Steinberg et al., 2022). Nonetheless, clinical phenotyping remains a cornerstone of risk assessment; the absence of identifiable pathogenic variants should not lead to an underestimation of risk, especially in patients presenting with multiple primaries or strong familial aggregation (Swetter et al., 2019).

The strengths of this study include the detailed and comprehensive phenotypic characterization of a well-defined high-risk cohort, as well as the stratification into clinically meaningful subgroups that are often analyzed together in previous studies. The use of a homogeneous clinical setting and standardized data collection further supports the internal validity of the findings. However, several limitations should be acknowledged. Its retrospective design may have introduced selection and information bias, particularly in the assessment of environmental exposures and the self-reported nature of the oncological family history, both of which may be subject to recall bias. Specifically, data regarding tanning bed use and sunburn history were entirely self-reported. Furthermore, the lack of granular quantitative data regarding the exact frequency or cumulative duration of artificial tanning precluded the possibility of conducting reliable sensitivity analyses for these variables. Additionally, the single-center setting may limit the generalizability of the findings to broader populations with different demographic and genetic backgrounds. Furthermore, genetic testing was not uniformly available across all patients, potentially leading to an underestimation of the true prevalence of germline susceptibility. Finally, the relatively limited sample size within specific subgroups may have reduced the statistical power to detect weaker associations, above all regarding phenotypic and environmental variables. Notably, the small size of the MPM+FM group (n = 63) significantly limited our statistical power. Consequently, several borderline non-significant findings, such as the trends observed for ulceration rates (p = 0.07), mitotic count (p = 0.10), or Fitzpatrick skin type III distribution (p = 0.07), could be the result of insufficient power. These variables may represent false-negative results rather than a true absence of association, meaning that these specific negative findings should be interpreted with caution.

From a clinical perspective, these findings support a more tailored approach to surveillance in high-risk melanoma patients. Individuals with sporadic MPM, particularly those with a late-onset disease, may benefit most from intensified dermatologic follow-up focused on early detection of additional primary tumors. Furthermore, patients with FM or combined phenotypes may require broader surveillance strategies that also consider associated malignancies and incorporate genetic counseling when appropriate, in line with current clinical management recommendations (Swetter et al., 2019). In this context, the integration of advanced diagnostic and staging modalities, such as high-resolution contrast-enhanced ultrasound for lymphatic mapping and refined indications for sentinel lymph node biopsy, plays a crucial role in the personalized management of these high-risk cohorts (Chan K et al., 2024; Rios-Sanchez MV et al., 2025). Given the rapidly evolving landscape of hereditary melanoma, scientific societies, including the Italian Melanoma Intergroup (IMI), are continuously working to periodically update and refine these inclusion criteria, ensuring an optimal and increasingly tailored patient selection.

The high prevalence of artificial ultraviolet exposure in familial cases further highlights the importance of targeted prevention strategies in younger, at-risk populations.

These findings reinforce the concept that high-risk melanoma is not a uniform entity but rather a spectrum of clinically and biologically distinct phenotypes. Sporadic multiple melanomas appear to be predominantly driven by cumulative exposure and demographic factors, whereas familial melanoma is characterized by earlier onset and broader cancer susceptibility. Integrating this phenotypic heterogeneity into clinical practice may improve personalized risk assessment, optimize surveillance strategies, and guide more targeted preventive approaches.

## Data Availability

The raw data supporting the conclusions of this article will be made available by the authors, without undue reservation.
